# BAR, SOFT AND DRI POST-HEPATIC TRANSPLANTATION: WHAT IS THE BEST FOR SURVIVAL ANALYSIS?

**DOI:** 10.1590/0102-672020210001e1576

**Published:** 2021-06-11

**Authors:** Fernando TORTEROLLI, Rafael Katsunori WATANABE, Fernando Issamu TABUSHI, Igor Luna PEIXOTO, Paulo Afonso Nunes NASSIF, Nertan Luiz TEFILLI, Sergio Luiz ROCHA, Osvaldo MALAFAIA

**Affiliations:** 1 Postgraduete Course in Principles of Surgery, Mackenzie Evangelical College of Paraná; 2 Mackenzie Evangelical College of Paraná, Curitiba, PR, Brazil; 3 Hospital São Vicente, Curitiba, PR, Brazil; 4 Federal University of Paraná, Curitiba, PR, Brazil

**Keywords:** Organ Dysfunction Scores, Liver Transplantation, Liver Cirrhosis, Survival Analysis, Severity of Illness Index, Escores de disfunção orgânica, Transplante de fígado, Cirrose hepática, Análise de sobrevida, Índice de gravidade de doença

## Abstract

**Background:**

Liver transplantation is the treatment of choice for patients with terminal liver disease. The Balance of Risk Score (BAR), Survival Outcomes Following Liver Transplantation (SOFT) and Donor Risk Index (DRI) scores are predictive systems for post-transplant survival.

**Aim:**

To evaluate the most accurate score and the best cutoff point for each predictor in the brazilian population.

**Method:**

Retrospective cross-sectional study of 177 patients. Data on the recipient, donor and transplant were analyzed and the prognostic scores BAR, SOFT and DRI were calculated for each transplant. To determine the BAR and SOFT cutoff points associated with death in three months, ROC curves were adjusted. Results : The best cutoff point for BAR was 9 points with an area under the ROC curve=0.69 and for SOFT it was 12 points with an area under the ROC curve=0.73. The DRI score did not discriminate survival (p = 0.139).

***Conclusion*:**

The SOFT score proved to be better than BAR for survival analysis post-hepatic transplantation and the DRI was not effective.

## Graphical abstract

[Fig f1a]

**Figure f1a:**
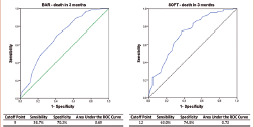
Ability of BAR and SOFT scores to predict death in three months’ time

[Fig f1as]

**Figure f1as:**
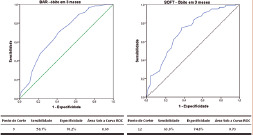
Habilidade dos escores BAR e SOFT em predizer o óbito em três meses

## RESUMO

**Racional:**

Transplante hepático é tratamento de escolha para pacientes com doença hepática terminal. Os escores Balance of Risk Score (BAR), Survival Outcomes Following Liver Transplantation (SOFT) e o Donor Risk Index (DRI) são sistemas preditores de sobrevida após o transplante.

**Objetivo:**

: Avaliar o escore de maior acurácia e o melhor ponto de corte de cada preditor na população brasileira.

**Método:**

: Estudo retrospectivo transversal de 177 pacientes. Foram analisados dados sobre o receptor, doador e o transplante e calculados os escores prognósticos BAR, SOFT e DRI para cada transplante. Para a determinar os pontos de corte de BAR e SOFT, associados a óbito em três meses, foram ajustadas curvas ROC.

**Resultados:**

: O melhor ponto corte para BAR foi 9 pontos com área sob a curva ROC=0,69 e para SOFT foi 12 pontos com área sob a curva ROC=0,73. O escore DRI não discriminou a sobrevida (p=0,139).

**Conclusão:**

O escore SOFT mostrou-se melhor do que o BAR para análise de sobrevida pós-transplante hepático, e o DRI não foi efetivo.

## INTRODUCTION

The liver transplantation is the chosen treatment to patients that have end stage liver disease ^1^. In the last couple of decades, there has been an expressive increase of these procedures. Between the years of 2008 and 2018, in Brazil, the number of cadaveric transplants has increased in 91.1% ^2^. In the American population, based on the United Network for Organ Sharing (UNOS), the survival of one year after the transplant can reach 89.7% ^3^, while in Brazil it reaches 75% ^4^.

The greatest obstacle for healing of end stage liver disease patients is the shortage of organs number. There is huge discrepancy between the demand and the number of transplants that are actually done ^2^. In Brazil in 2018 the mortality rate on waitlists was 45.7% ^2^. Due to that, one of the strategies has been the use of donors known as non - ideals, adjacent, with expanded criteria or bordering, in search of increasing the number of transplants and decreasing the time spent on waitlists ^5^ ^,^ ^6^_._ Yet, the rate of mortality on this list is high ^2,7^.

In Brazil, until 2006, the base to allocating cadaveric livers was the time on waitlist, except for patients who had fulminant hepatitis or needed an emergency retransplatation ^8^. Nowadays, more consistent criteria are available. Using the score Model for End-Stage Liver Disease (MELD) we can estimate the severity of the cirrhotic patient and the mortality rate on waitlist, and recently this has been the base for the organ allocating system ^9^ ^,^ ^10^.

Hoping to enhance even more the standards the prognosis scores Balance of Risk Score (BAR), Survival Outcomes Following Liver Transplantation (SOFT) and Donor Risk Index (DRI) were created, estimating survivability after transplant individually, according to the characteristics of the graft and the receiver ^11^ ^-^ ^14^. The literature related to these predictors is scarce and not widespread, which leads to big medical centers, sometimes, not knowing these scores which could be of great importance for a better indication of transplant. Not only would the patient’s case severity be decisive ^15^, but also the survivability estimated after the procedure, which directly depends on the organ available.

The BAR, SOFT and DRI scores were developed based on american and european populations ^11^ ^-^ ^13^. In Brazil, particularly, transplant centers that use these scores prognosis’ system are yet unknown. So, studies that enable a better evaluation when used on the brazilian population become highly necessary.

The main goal of this presented study was to evaluate among the three scores which is more accurate and the better cut point for the brazilian population. 

## METHODS

This is a retrospective and cross-sectional observational study, based on prospective data, done in one center, with 177 cadaveric liver transplants, done in the Liver Transplantation Unit of Hospital São Vicente*,* in the city of Curitiba PR, Brazil, in the period between June 16^th^ , 2016 and August 9^th^ , 2018.

All of the patients that underwent the liver transplant who were 18 or more years old were included and obtained from the service database. The exclusion criteria were patients who were under 18 years old, multiple organ transplant cases, and the ones which the information on the database was not enough to fulfill the Liver Transplant Report, which were not observed on the studied sample universe. Data was collected from the hospital charts, and information from the donors were supplied by the National Center of Transplants (CNT).

The pre-surgical variables studied for the receivers were: diagnoses, weight (kg), height (m), BMI (body mass index), gender, age, race, the number of previous transplants, life support, previous abdominal operation in the upper abdomen, pre-transplant dialyses, pre-transplant ICU hospitalization, pre - transplant infirmary hospitalization, hepatic encephalopathy, ascites, upper gastrointestinal bleeding 48 hours pre - transplant, pre - transplant portal-vein thrombosis, albumin, laboratory MELD and adjusted MELD.

The data concerning the donor were: age, height (cm), cause of death, race, creatinine (mg/dl), days in the ICU and if it was donation after cardiac death. The data that referred to the transplant were: date of the procedure, date of death, location of the donation, split liver, surgical mortality (30 days after the procedure), time of cold ischemia (hours), post-operation complications in 30 days (Clavien-Dindo classification), BAR, SOFT and DRI scores.

Based on the data collected in each transplant, the scores prognosis BAR, SOFT and DRI were calculated, for each one of them. The calculation of BAR and DRI were done on October 2^nd^, 2018, through the use of online calculators available on https://www.assessurgery.com/bar-score/bar-score-calculator/ and https://gastro.cchmc.org/calculators/donor-risk-index/, respectively ^16^ ^,^ ^17^. The SOFT score was calculated according to the “Table 4: PSOFT and SOFT scores” elaborated by Rena et al. ^11^.

To calculate the survival of each patient the date of the transplant was considered and, in case of death, when it happened. To the patients that did not have reported death, it was considered the date of the last follow up on March 21^st^, 2019. All of the transplanted patients on the service do routine post-operation follow ups

### Statistical analysis

The results were described by averages, standard deviation, minimum and maximum values (quantitative variables) or by frequencies and percentages (categorical variables). The association amongst quantitative variables was analyzed, estimating the Spearman correlation coefficient. In order to describe the time of survival, the Kaplan-Meier survival curves were used. The estimated measurement of association was hazard ratio (HR) to which were presented a confidence interval of 95%. To determine the BAR and SOFT cutoff points, associated to death in three months, the Reciver Operating Characteristic (ROC) curves were adjusted. Values of p<0,05 indicate statistic significance. The data was analyzed using the computational program Stata/SE v.14.1. StataCorpLP, USA.

## RESULTS

Among the 177 transplant patients, 128 (72.3%) were men and 49 (27.7%) were women. The age of the receivers was 56±11.1 (19-77) years old. The patients’ laboratory MELD was 22.5±8.4 (7-53) points. It was verified that six (3.4%) of the patients required retransplant. From the total, 21 (11.9%) presented pre-transplant portal vein thrombosis (Table 1).

The location of capture of most of the grafts (57.6%) was inside of the state itself; 28.2% was in local origin (up to 40 km from the transplant center) and 14.1% were captured out of the state. The average of cold ischemia time of the graft was 5.7±1.6 (2.5-14) hours, from which 144 (64.4%) had a time less or equal to 6 hours (Table 1)

The average of the BAR score of the studied population was 9.0±4.6 (1-24), and the SOFT score was 11.3±9.4 (0-42). The average found to DRI was 1.5±0.4 (0.9-2.6, Table 1).

**TABLE 1:** Descriptive characteristics of the receiver, operative factors and the BAR, SOFT and DRI scores.

Variable	Rating	Outcome*
Age (years)		56±11.1 (19-77)
(3 intervals)	≤40	16 (9.0)
	41 a 60	94 (53.1)
	>60	67 (37.9)
Gender	Female	49 (27.7)
	Male	128 (72.3)
BMI		25.6±3.9 (18.4-51.4)
(3 intervals)	≤25	92 (52.0)
	25.1 a 30	71 (40.1)
	>30	14 (7.9)
Laboratory MELD		22.5±8.4 (7-53)
(4 intervals)	6 a 15	26 (14.7)
	16 a 25	104 (58.8)
	26 a 35	33 (18.6)
	>35	14 (7.9)
Ajusted MELD		23.5±7.6 (11-53)
(4 intervals)	6 a 15	12 (6.8)
	16 a 25	117 (66.1)
	26 a 35	34 (19.2)
	>35	14 (7.9)
Retransplantation	No	171 (96.6)
	Yes	6 (3.4)
Pre-transplant portal vein thrombosis	No	156 (88.1)
	Yes	21 (11.9)
Organ Location	Inside of the state	102 (57.6)
	Local	50 (28.2)
	Out of the state	25 (14.1)
Cold ischemia time (hours)		5.7±1.6 (2.5-14)
(2 intervals)	≤6	114 (64.4)
	>6	63 (35.6)
Scores of BAR, SOFT and DRI		
BAR		9.0±4.6 (1-24)
(2 intervals)	≤9	111 (62.7)
	>9	66 (37.3)
SOFT		11.3±9.4 (0-42)
(2 intervals)	≤12	115 (65)
	>12	62 (35)
DRI		1.5±0.4 (0.9-2.6)
(5 intervals)	≤1.2	31 (17.5)
	1.21 a 1.4	39 (22)
	1.41 a 1.6	38 (21.5)
	1.61 a 1.8	30 (16.9)
	>1.8	39 (22)

*Result described by average ± standard deviation (minimum-maximum) or by frequency (percentage)

**FIGURE 1:**
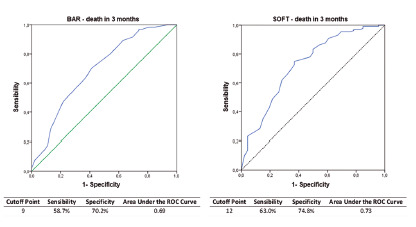
ROC curves applied to the BAR and SOFT scores’ abilities of predicting death in three months.

The cutoff points defined for BAR and SOFT scores were 9 and 12 points, respectively. The sensibility, specificity and area under the ROC curve in the best cutoff point of each score, to the population sample, are represented in Figure 1, and both are statistically significant (p<0.001) in foretelling three month mortality. An area under the curve ROC>0.7 indicates an useful test and if >0.8 an excellent test ^18^ ^,^ ^19^. The SOFT score was the only one able to reach the area under the curve of ROC >0.7 to foretell the mortality in three months, meaning it is a clinically useful test. A cutoff point to foretell three month mortality for the DRI score could not be defined, because it did not discriminate the post-transplant mortality (p=0.139).

**FIGURE 2:**
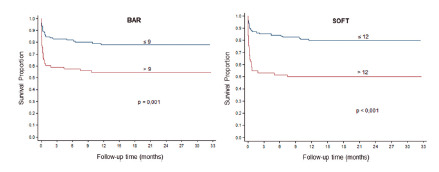
Estimated survival through the Kaplan-Meier curves of the patients who had BAR ≤9 and BAR >9, and SOFT ≤12 and SOFT >12

The survival analysis estimated for patients with BAR ≤9 was, at the end of three months’ time, of 82.9% vs. 59.1% with BAR >9 (p=0.001). At the end of twelve months to patients with BAR ≤9 was of 73.9% vs. 51.6% with BAR >9 (p=0.001, Figure 2). The estimated survival of patients with SOFT ≤12 was, at the end of three months, of 85.2% vs. 53.2% with SOFT >12 (p<0.001). At the end of 12 months to patients with SOFT ≤12 was of 79.7% vs. 50% with SOFT >12 (p<0.001, Figure 2).

Analyzing the correlation of the three scores with the patients’ laboratory MELD, BAR (p<0.001), SOFT (p<0.001) and DRI (p=0.006) were significantly correlated; however, the coefficient of the Spearman correlation showed a better association between laboratory MELD and BAR (r=0.74, Table 2).

**TABLE 2:** Spearman correlation and values of p from statistical tests among MELD and BAR, SOFT e DRI prognosis scores.

Scores	n	Spearman’s correlation coefficient	p
Laboratory MELD x BAR	177	0.74	<0.001
Laboratory MELD x SOFT	177	0.53	<0.001
Laboratory MELD x DRI	177	-0.21	0.006

The evaluation of the association between the variables and general survival after transplant is presented on Table 3. Variables, which had more than two classifications, had one defined as reference (ref) and the other ones were compared to that one. Determinants to survival after transplant were the following variables: BAR score >9 (HR 2.58; CI 95% 1.50-4.41; p=0.001), SOFT score >12 (HR 3.22; CI 95% 1.97-5.53; p <0.001), woman (HR 2.00; CI 95% 1.16-3.46; p=0.013), laboratory MELD >35 points (HR 7.56; CI 95% 2.58-22.2; p<0.001) and retransplanted patients (HR 4.75; CI 95% 1.71-13.2; p=0.003, Table 3).

**TABLE 3:** Association between demographic variables, clinical variables and scores and the after transplant survival.

Variable	Rating	% Death	p*	HR (CI 95%)
Age (years)	≤40 (ref)	37.5		
	41 a 60	22.3	0.184	0.54 (0.22-1.34)
	>60	40.3	0.885	1.07 (0.44-2.59)
Gender	Male (ref)	25.8		
	Female	42.9	0.013	2.00 (1.16-3.46)
BMI	≤25 (ref)	32.6		
	25.1 a 30	31.0	0.770	0.92 (0.53-1.60)
	>30	14.3	0.238	0.42 (0.10-1.77)
Laboratory MELD	6 a 15 (ref)	19.2		
	16 a 25	25.0	0.566	1.32 (0.51-3.45)
	26 a 35	39.4	0.122	2.26 (0.80-6.34)
	>35	71.4	<0.001	7.56 (2.58-22.2)
Retransplantation	No (ref)	29.2		
	Yes	66.7	0.003	4.75 (1.71-13.2)
Pre-transplant portal vein trombosis	No (ref)	28.8		
	Yes	42.9	0.143	1.71 (0.83-3.49)
Cold ischemia time (hours)	≤6 (ref)	27.2		
	>6	36.5	0.168	1.46 (0.85-2.51)
DRI	≤1.2 (ref)	32.3		
	1.21 a 1.4	30.8	0.898	0.95 (0.41-2.19)
	1.41 a 1.6	18.4	0.170	0.51 (0.19-1.34)
	1.61 a 1.8	46.7	0.280	1.56 (0.69-3.52)
	>1.8	28.2	0.655	0.82 (0.35-1.94)
BAR	≤9 (ref)	21.6		
	>9	45.5	0.001	2.58 (1.50-4.41)
SOFT	≤12 (ref)	20.0		
	>12	50.0	<0.001	3.22 (1.97-5.53)

*Values of p<0.05 indicate statistical significance; Hazard ratio (HR) with confidence interval (CI) of 95%; ref=reference

## DISCUSSION

The high number of deaths of patients who are on the waitlist in Brazil is ^2^, in some parts, a reflex of the low rates of organ donation, around 16.6 per million population (pmp) ^4^, while in the USA and other european countries the rates go up to 25 and 30 pmp, respectively ^20^. Clearly, that increases the time and mortality of patients on the waitlist, and because of that, surgeons end up using expanded criteria livers ^7^. The survival of one year reached in American services is 89.7% ^3^, while in brazilian reaches 75% ^4^. On the studied sample the average laboratory MELD of patients was 22.5±8.4 (7-53) points, which sets a high degree of gravity of the patients and consequently, a shorter survivability ^10,21,22^ . Beyond that, in the study the average DRI of grafts was 1.5±0.4 (0.9-2.6), which may indicate quality of grafts beneath the ideal ^9^ ^,^ ^21^ ^,^ ^23^. It is clear the difficulty faced by brazilian surgeons on the current scenario. Having a foretelling survival score after transplant would be of great help, since the conditions faced are difficult.

Several studies referring to new survival foretellers are being developed to help on the decision making process of liver transplantion ^11^ ^-^ ^13^ ^,^ ^19^ ^,^ ^24^. However, up to now the big centres use and recognize only the foreteller MELD as clinically useful, northing all of the allocation process of the grafts. The MELD score is able to predict the mortality in a three months period of the patient on the waitlist ^9^ ^,^ ^10^ ^,^ ^25^ ^,^ ^26^. However, while trying to use it as a survival foreteller three months after the transplant, it reveals to be inefficient ^11^ ^,^ ^12^ ^,^ ^22^.

Dutkowski et al. ^12^ developed a survival foreteller after transplant BAR that analyses six variables in total, from both the receiver and the donor, and it presented a good accuracy in foretelling the three month-after transplant-survival. They obtained an area under the ROC curve of 0.7 with a cutoff of 18 points, since they noticed that from it, the survival started to deteriorate exponentially. When applying the BAR score on the studied sample, the test also revealed itself useful to foretell the death in a three-month period (p<0.001). The best cutoff point determined to the sample was of 9 points (sensibility =58.7% and specificity =70.2%), with an area under the ROC curve of 0.69, not reaching the parameters established by international consensus to be considered as a clinically useful trial.

In a brazilian study, Campos Junior et al. ^27^ used the BAR score in 402 transplanted patients samples and got to the conclusion that it is a good foreteller of the survival in a three and twelve months period. The best cutoff point was of 11 points (sensibility =39% and specificity =87%) and the area beneath the ROC curve of 0.65, also being under international consensus as clinically useful. It was verified in the present study an area under the ROC curve superior to the ones found by Campos Junior et al. ^27^ and Åberg et al. ^28^ who had similar results, which suggests a superior performance of the score on the studied sample. 

The different cutoff points found in the literature for the BAR foreteller showed that it varies depending on the population it is applied on. There is the need of a bigger number of studies in order to achieve a consensus on what is necessary to change and also the best cutoff point, before implementing them on the brazilian services. Dutkowski et al. ^12^ initially developed the score based on american and european populations, and it is noticeable that when it was applied on the brazilian population, there was a difference in the results, this fact was also observed by Campos Junior et al. ^27^. It is demonstrated that the score must go through adaptations before being applied onto the brazilian reality.

The SOFT score also used to estimate the survival after transplant, developed by Rana et al. ^11^ establishes 22 relevant variables. They found an area under the ROC curve of 0.7 when analyzing the survival in three months. A cutoff point was established, by another author, as 15 points, which also verified in his sample an area beneath the ROC curve of 0.7 ^12^.

On this present study the SOFT score had satisfactory results when discriminating the three months death (p<0.001). The area under the ROC curve was of 0.73, even superior to the one found in other studies ^11^ ^,^ ^12^. The best cutoff point verified on the analyzed sample was of 12 points (sensibility = 63.0% and specificity =74.8%), inferior when compared to the one established by Dutkowski et al. ^12^, which was 15 points. Then, it makes it possible to confirm once again the hypotheses that before implanting the score in a different population from where it was first developed, the score should go through some adaptations.

In another study, Feng et al. ^13^ established the DRI score, with eight variables exclusive to the donor as relevant to foretell the risk of primary failure of the graft and foretell the survival after three months. When applying this score, Dutkowski et al. ^12^ verified the area under the ROC curve of only 0.5 to three months. The same was observed by Åberg et al. ^28^ that also verified the area between 0.5-0.65 when foretelling the survival in one year. On the sample that was studied here, the DRI score was not able to discriminate the mortality in three months after transplant (p=0.139), making it impossible to calculate the area beneath the ROC curve and define a cutoff point.

Among the three applied scores on the sample, the SOFT score had the biggest accuracy in foretelling death in three months, opposite to Dutkowski et al. ^12^, that observed it to be the BAR score. One of the visible disadvantages of the SOFT score is the 22 variables raised in relation to the receiver and donor, compared to the six ones needed on BAR.

It is clear that gathering variables from the receiver as well as from the donor (BAR and SOFT) leads to a best accuracy of the prognosis model in foretelling the survival when compared to the ones that analyze only the donor’s variables (DRI).

When studying the correlation among the three prognosis scores compared to the laboratory MELD score of the patients, all of them are significantly correlated with MELD; however, the best association degree was with BAR (r=0.74), which was also described by Dutkowski et al. ^12^.

It was possible to observe a shorter survival after transplant to the female gender variable (p=0.013), differently from obtained by Rana et al. ^11^ and Dutkowski et al. ^12^ that dismissed the variable as a risk factor. There was also short survival of previous transplant patients (p=0.003), as established priorly by other studies ^11^ ^,^ ^12^ and laboratory MELD >35 points (p<0.001), which corroborates partially with the literature, that presumes values even smaller than 35 points do increase the risk of death ^11^ ^,^ ^12^.

## CONCLUSION

Among the analyzed prognosis scores, SOFT had greater accuracy in foretelling death in three months’ time, and overcame established demands to be a clinically useful trial. The best cutoff point in the studied population for BAR was 9 points. To SOFT, the cutoff point was 12. When correlating the prognosis scores with the laboratory MELD, the biggest correlation was with BAR. It was verified that the female gender, previous transplant and MELD >35 points are associated with a shorter survival.

## References

[1] Fox AN, Brown RS (2012). Is the Patient a Candidate for Liver Transplantation. Clin Liver Dis.

[2] Associação Brasileira de Transplante de Órgãos (ABTO) (2018). Registro Brasileiro de Transplantes - Dimensionamento dos Transplantes no Brasil e em cada estado (2011-2018) [Internet].

[3] Meirelles RF, Salvalaggio P, Bruno de Rezende M, Silva Evangelista A, Della Guardia B, Eduardo Lourenço Matielo C (2015). Liver transplantation: history, outcomes and perspectives. Einstein.

[4] Associação Brasileira de Transplante de Órgãos (ABTO) (2017). Registro Brasileiro de Transplantes - Dimensionamento dos Transplantes no Brasil e em cada estado (2010-2017) [Internet].

[5] Rocha MB, Boin IFSF, Escanhoela CAF, Leonardi LS (2004). Can the use of marginal liver donors change recipient survival rate. Transplant Proc.

[6] Freitas ACT de, Coelho JCU, Watanabe MR, Lima RL das C (2020). Relationship between donor quality and recipient gravity in liver transplant. Arq Bras Cir Dig.

[7] Silveira F, Silveira FP, Macri MM, Nicoluzzi JEL (2012). Análise da mortalidade na lista de espera de fígado no Paraná, Brasil. O que devemos fazer para enfrentar a escassez de órgãos. ABCD Arq Bras Cir Dig.

[8] Secretaria de Assistência à Saúde do Ministério da Saúde do Brasil (1998). Portaria GM no 3.407 de 5 de agosto de 1998.

[9] Wiesner R, Edwards E, Freeman R, Harper A, Kim R, Kamath P (2003). Model for end-stage liver disease (MELD) and allocation of donor livers. Gastroenterology.

[10] Manduca Palmiero HO, Kajikawa P, Boin IFSF, Coria S, Pereira LA (2010). Liver recipient survival rate before and after model for end-stage liver disease implementation and use of donor risk index. Transplantation Proceedings.

[11] Rana A, Hardy MA, Halazun KJ, Woodland DC, Ratner LE, Samstein B (2008). Survival Outcomes Following Liver Transplantation (SOFT) Score: A Novel Method to Predict Patient Survival Following Liver Transplantation. Am J Transplant.

[12] Dutkowski P, Oberkofler CE, Slankamenac K, Puhan MA, Schadde E, Müllhaupt B (2011). Are There Better Guidelines for Allocation in Liver Transplantation. Ann Surg.

[13] Feng S, Goodrich NP, Bragg-Gresham JL, Dykstra DM, Punch JD, DebRoy MA (2006). Characteristics associated with liver graft failure: the concept of a donor risk index. Am J Transplant.

[14] Assis BS de, Coelho FF, Jeismann VB, Kruger JAP, Fonseca GM, Cecconello I (2020). Total laparoscopic vs. open liver resection: comparative study with propensity score matching analysis. Arq Bras Cir Dig.

[15] Malinowski EA, Matias JEF, Percicote AP, Nakadomari T, Robes R, Petterle RR (2020). Conservation of both hematocrit and liver regeneration in hepatectomies: A vascular occlusion approach in rats. Arq Bras Cir Dig.

[16] AssesSurgery GmbH (2019). Online BAR Score Calculation [Internet].

[17] Feng S, Goodrich N P, Bragg-Gresham J L, Dykstra D M, Punch J D, DebRoy M A, Greenstein S M, Merion R (2007). Donor Risk Index for Liver Transplantation Calculator [Internet].

[18] Hanley JA, McNeil BJ (1982). The meaning and use of the area under a receiver operating characteristic (ROC) curve. Radiology.

[19] Jacob M, Lewsey JD, Sharpin C, Gimson A, Rela M, van der Meulen JHP (2004). Systematic review and validation of prognostic models in liver transplantation. Liver Transplant.

[20] Pacheco L (2016). Transplante de fígado no Brasil. Revista do Colégio Brasileiro de Cirurgiões.

[21] Boin IDFSF, Leonardi MI, Udo EY, Sevá-Pereira T, Stucchi RSB, Leonardi LS (2008). Aplicação do escore MELD em pacientes submetidos a transplante de fígado: Análise retrospectiva da sobrevida e dos fatores preditivos a curto e longo prazo. Arq Gastroenterol.

[22] Brandão A, Fuchs SL, Gleisner AL, Marroni C, Zanotelli ML, Cantisani G (2009). MELD and other predictors of survival after liver transplantation. Clin Transplant.

[23] Schlitt HJ, Loss M, Scherer MN, Becker T, Jauch KW, Nashan B (2011). Current developments in liver transplantation in Germany: MELD-based organ allocation and incentives for transplant centres. Z Gastroenterol.

[24] Anastácio LR, Ferreira LG, Ribeiro HS, Diniz KGD, Lima AS, Correia MITD (2019). Sarcopenia, obesity and sarcopenic obesity in liver transplantation: A body composition prospective study. Arq Bras Cir Dig.

[25] Bernardi M, Gitto S, Biselli M (2011). The MELD score in patients awaiting liver transplant: Strengths and weaknesses. Journal of Hepatology.

[26] Freitas ACT de, Itikawa WM, Kurogi AS, Stadnik LG, Parolin MB, Coelho JCU (2010). The impact of the model for end-stage liver disease (MELD) on liver transplantation in one center in Brazil. Arq Gastroenterol.

[27] Campos ID de, Stucchi RSB, Udo EY, Boin I de FSF (2015). Application of the BAR score as a predictor of short- and long-term survival in liver transplantation patients. Hepatol Int.

[28] Åberg F, Nordin A, Mäkisalo H, Isoniemi H (2015). Who is too healthy and who is too sick for liver transplantation: External validation of prognostic scores and survival-benefit estimation. Scand J Gastroenterol.

